# Variability in the lymph node retrieval after resection of colon cancer: Influence of operative period and process

**DOI:** 10.1097/MD.0000000000004199

**Published:** 2016-08-07

**Authors:** Jung Pil Choi, In Ja Park, Byung Cheol Lee, Seung Mo Hong, Jong Lyul Lee, Yong Sik Yoon, Chan Wook Kim, Seok-Byung Lim, Jung Bok Lee, Chang Sik Yu, Jin Cheon Kim

**Affiliations:** a Department of Surgery, Dong Kang Medical Center, Ulsan; b Division of Colon and Rectal Surgery, Department of Surgery, University of Ulsan College of Medicine, Asan Medical Center; c Department of Pathology, University of Ulsan College of Medicine, Asan Medical Center; d Department of Clinical Epidemiology and Biostatistics, University of Ulsan College of Medicine, Asan Medical Center, Seoul, Korea.

**Keywords:** colon neoplasm, lymph node retrieval, multidisciplinary

## Abstract

The purpose of this study was to evaluate variations in the number of retrieved lymph nodes (LNs) over time and to determine the factors that influence the retrieval of <12 LNs during colon cancer resection.

Patients with colon cancer who were surgically treated between 1997 and 2013 were identified from our institutional tumor registry. Patient, tumor, and pathologic variables were evaluated. Factors that influenced the retrieval of <12 LNs were evaluated using multivariate logistic regression modeling, including time effects.

In total, 6967 patients were identified. The median patient age was 61 years (interquartile range [IQR] = 45–79 years) and 58.4% of these patients were male. The median number of LNs retrieved was 21 (IQR = 14–29), which increased from 14 (IQR = 11–27) in 1997 to 26 (IQR = 19–34) in 2013. The proportion of patients with ≥12 retrieved LNs increased from 72% in 1997 to 98.8% in 2013 (*P* < 0.00001). This corresponded to the more recent emphasis on a multidisciplinary approach to adequate LN evaluation. The number of retrieved LNs was also found to be associated with age, sex, tumor location, T stage, and operative year. Tumor location and T stage influenced the number of retrieved LNs, irrespective of the operative year (*P* < 0.05). Factors including a tumor location in the sigmoid/left colon, old age, open resection, earlier operative year, and early T stage were more likely to be associated with <12 recovered LNs (*P* < 0.5; chi-squared test) (*P* < 0.001).

The total number of retrieved LNs may be influenced by tumor location and T stage of a colon cancer, irrespective of the year of surgery. LN retrieval after colon cancer resection has increased in recent years due to a better awareness of its importance and the use of multidisciplinary approaches.

## 1 Introduction

Approximately 75% of patients with colorectal cancer will present with potentially curable disease by surgical resection,^[1]^ including en bloc resection of the associated mesentery by proximal ligation at the origin of the primary feeding vessels. The majority of colon cancer-related deaths are attributed to advanced, recurrent, or metastatic disease. In the absence of a distant metastasis, the regional lymph node (LN) status is the most important pathological predictor of long-term survival in patients with colon cancer. In addition, the LN status is the key consideration when deciding to adjuvant chemotherapy.^[2,3]^ Hence, pathological evaluations of patients with colon cancer should consist of a complete assessment of the LNs contained within the resected specimen. The number of metastatic LNs identified may be influenced by the total number of LNs examined, thus increasing the probability of stage migration.^[4–6]^

Some studies have reported that increasing the number of LNs evaluated at the time of curative resection in patients with colon cancer is associated with improved survival.^[4,7,8]^ The number of LNs retrieved from a patient with colon cancer has been identified as a potentially important measure of the quality of cancer care by many organizations, including the American College of Surgeons, the American Society of Clinical Oncology, the National Comprehensive Cancer Network (NCCN), various health insurance providers, and others. A minimum of 12 examined LNs at curative resection is now advocated as a quality measure^[9]^ and is recommended for proper staging. Despite these recommendations, however, a previous national cancer database study that evaluated colectomies performed in nearly 1300 hospitals reported that only 37% of the studied cases achieved at least 12 LNs^[10]^ and that the median number of recovered LNs between 1988 and 2000 in the United States was 9.^[11]^

The number of LNs examined at the time of colectomy can vary widely according to the surgical technique, pathological examination method, and tumor and patient factors such as tumor location, age, obesity, and immune response.^[10,12–15]^ The process of evaluating LNs in colon cancer has also changed over time and it can differ between attending surgeons or pathologists. Using tumor registry data from our single institution, we evaluated the changes of total number of harvested lymph nodes and the proportion of patients with colon cancer who had yielded <12 LNs over the study period. Moreover, we analyzed factors that influenced the number of yielded LNs in resected colon cancer specimens.

## 2 Materials and methods

### 2.1 Patients and identification

This retrospective cohort study was performed on all patients in whom a nonmetastatic adenocarcinoma of the colon was surgically treated between 1997 and 2013 at the University of Ulsan College of Medicine and had been included in the Asan Medical Center tumor registry. Data on demographics (age, sex, race, and year of diagnosis), tumor-related variables (primary site, histologic classification, and grade), and pathologic variables (TNM stage, total number of involved LNs, and total number of examined LNs) were collected. Changes in clinical practice such as introduction of multidisciplinary approaches in 2005 were also included in the analysis. The multidisciplinary team approach has been adopted in our hospital as part of the treatment planning in the clinic and in reviews of radiologic, surgical, and pathological results at regular seminars. The American Joint Commission on Cancer (AJCC) staging system (7th edition) was applied for TNM staging in our study patients. This staging system has been revised many times, however, and our study patients were staged according to the system that was in use in the year of the surgery. In order to compare the number of LNs yielded using equivalent definitions of T and N stage, we staged these patients again in accordance with the most recent AJCC 7th edition. This study was approved by the institutional review board of Asan Medical Center and the requirement for informed consent was waived due to the retrospective nature of the analysis.

### 2.2 Surgical method and pathologic lymph node evaluation

The surgical approach to colon cancer resection at our hospital incorporates 3 main principles: ligation of feeding vessels at their roots, principal node removal, and obtaining sufficient resection at both the proximal and distal margins. For lesions located in the right colon and proximal- to mid-transverse colon, a right hemicolectomy including a middle colic artery ligation at its root has been recommended. Cancers located at the distal transverse to the descending colon were treated using a left hemicolectomy. Anterior resections were performed for sigmoid colon cancer. Transverse colectomies were not usually performed. A lateral approach was used for open resection. Laparoscopic approaches were introduced in 2002 at our institution and have been performed in up to 50% of patients with colon cancer since 2010. For laparoscopic resections, the medial to lateral approach was usually taken. The principle of radical resection was same with that of open resection. Most of the operations were undertaken by 3 to 5 experienced colorectal surgeons. Some of the surgeries were done by colorectal fellows.

The pathologic lymph node examination technique at our hospital did not change during our study period. Briefly, the resected specimens were received fresh or minimally fixed in formalin. Mesenteric lymph nodes were initially harvested by manual node dissection, with serial sectioning, visualization, and palpation of the mesenteric tissue, followed by routine processing of the identified lymph nodes. Dedicated pathologists with expertise in gastrointestinal malignancies were responsible for lymph node evaluation.

### 2.3 Statistical analysis

Patient demographics and tumor-related variables were determined from the medical records. All of the data collected for this study were summarized as a mean ± standard deviation for continuous variables or frequency (percentage) for categorical data. To evaluate the risk and prognostic factors that affected the number of recovered LNs, the multiway analysis of variance model was applied. The factors that affected the retrieval of <12 LNs were evaluated by multivariate logistic regression modeling, which included time effects. All analyses were conducted using SAS software (version 9.4; Nary, NC).

## 3 Results

### 3.1 Patient characteristics

We identified 6967 patients with colon cancer from the database who met the study criteria. The median patient age was 62 years (interquartile range [IQR] = 55–79 years) and men were in the majority (58.4%). Tumors were right-sided in 38.8% of these patients and left-sided in 54% of patients. The tumor stage according to the AJCC 7th edition was I in 19.6% of patients, II in 45.9%, and III in 34.5%. Throughout the study period, 96.7% of the resections at our hospital were performed by a surgeon who specialized in colorectal cancer surgery. The characteristics of the study patients are listed in Table 1.

**Table 1:**
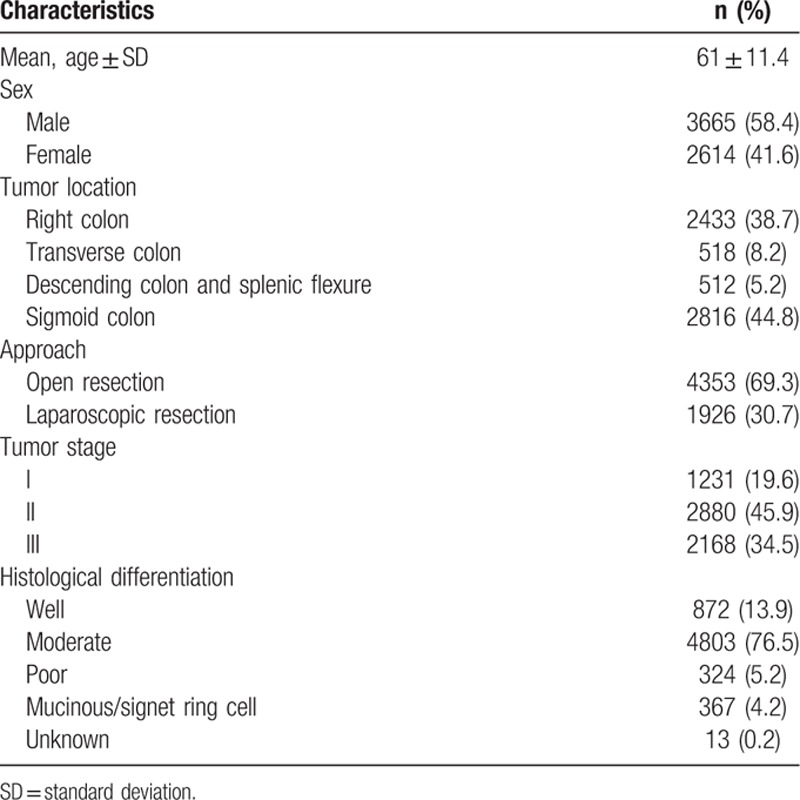
Patient and tumor characteristics (n = 6967).

### 3.2 Changes in nodal evaluation over time

**Figure 1:**
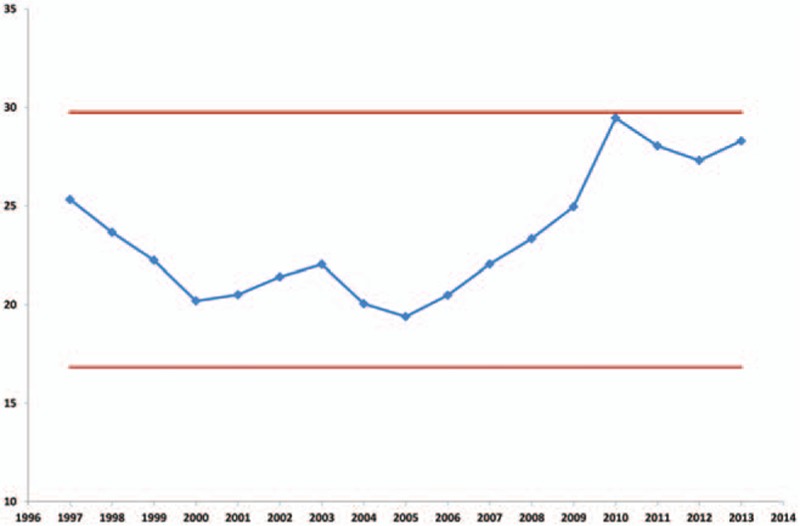
Control chart of the median number of lymph nodes and interquartile range examined by operative year.

**Figure 2:**
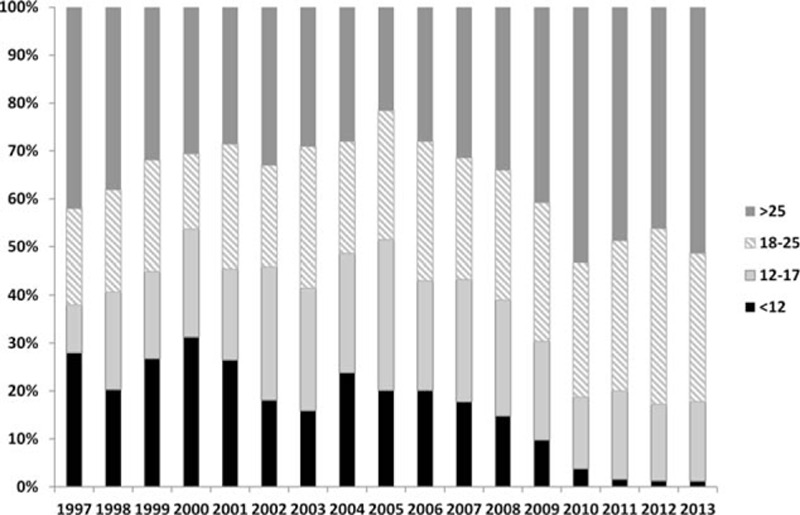
Percentage of retrieved lymph nodes by year.

The median number of LNs examined in our study cohort was 22 (IQR = 15–29). This number varied over time and increased from 16 (IQR = 8–28) in 2000 to 26 (IQR 19–35) in 2013 (*P* < 0.0001) (Fig. 1). Consequently, the proportion of patients with <12 LNs examined decreased over this period, from 28% in 1997 to 1.2% in 2013 (*P* < 0.00001) (Fig. 2). The number of LNs evaluated demonstrated 2 plateaus: one during 2004 to 2008, and the other from 2010 to 2013. The number of retrieved LNs was quite variable according to the tumor location over time. This variability was most prominent for transverse colon cancer. The recovered number of LNs also differed according to pT stage and was greater for pT3 and T4 disease. However, this has stabilized since 2010.

### 3.3 Factors related to the number of recovered LNs

**Figure 3:**
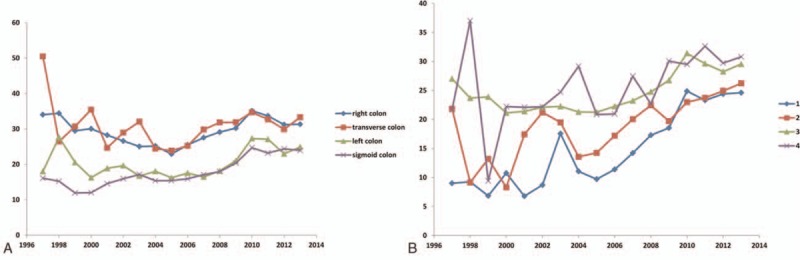
Influence of (A) tumor location and (B) pT stage on the number of lymph nodes recovered according to the operative year.

The number of retrieved LNs was found to be associated with the tumor location within the colon (*P* < 0.0001), age (*P* < 0.001), sex (*P* = 0.002), operative year (*P* < 0.001), and stage (*P* < 0.001), but was independent of the operator (Table 2). Over time, the number of retrieved LNs for patients with pT1/2 disease increased and the difference from patients with pT3/4 disease decreased (Fig. 3). The clinicopathological variables associated with the number of retrieved LNs also changed over time. Considering this change, we found that the tumor location and stage influenced the number of retrieved LNs during the operative year. This suggests that the variability in the number of retrieved LNs according to location of tumor and stage changed over time. Tumors located in the left and sigmoid colon were more likely to be associated with the retrieval of fewer LNs in comparison with tumors in the right or transverse colon. Multivariate analysis including the influence of operative year was done to identify factors associated with retrieval of <12 LNs. Old age, early cancer stage, the operative approach (i.e., open approach), and the year of operation showed an association with the retrieval of <12 LNs (Table 3).

**Table 2:**
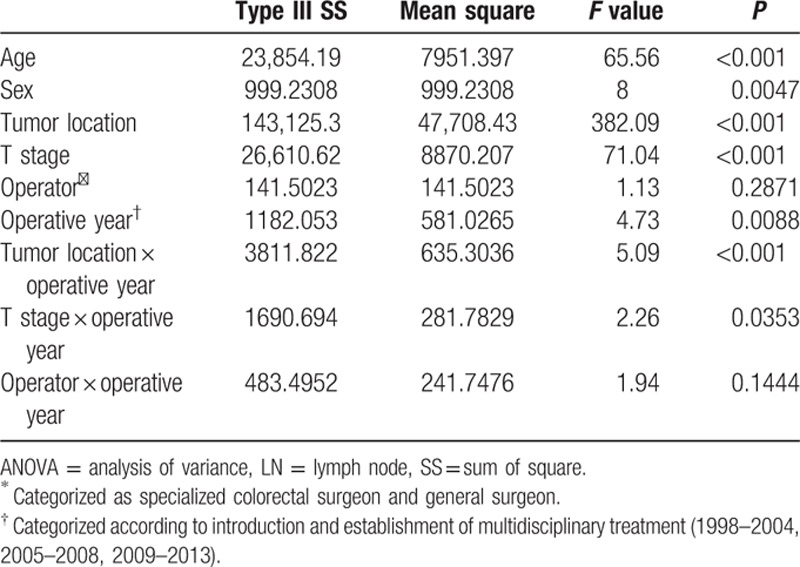
Factors associated with the number of recovered LNs, ANOVA table.

**Table 3:**
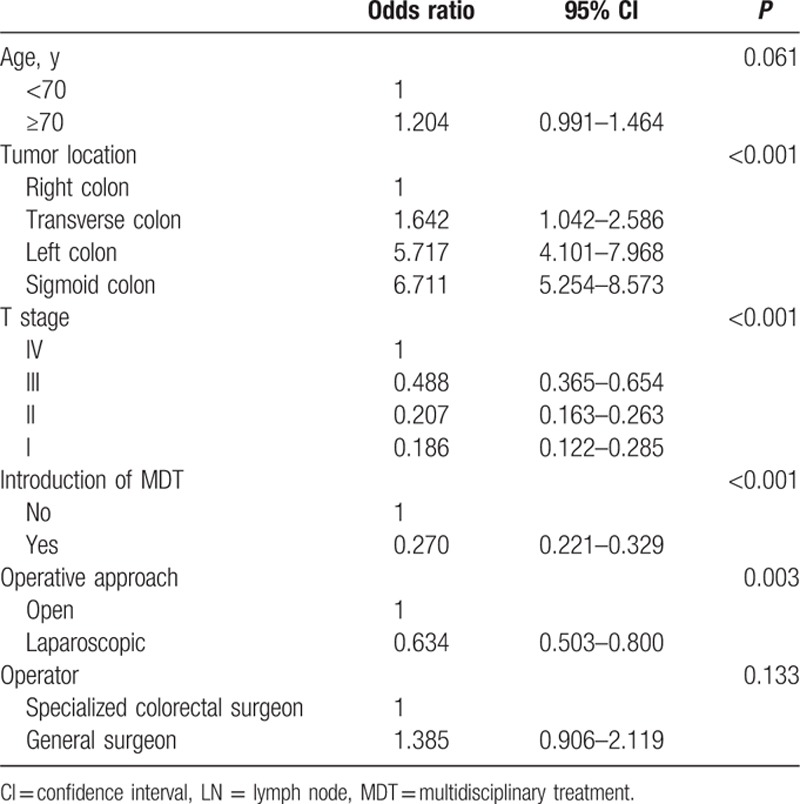
Factors associated with <12 recovered LNs: multivariate analysis.

## 4 Discussion

We have found in our present study that the retrieved number of LNs for colon cancer differs according to a variety of clinicopathological features such as age, sex, tumor location, pathological stage, and operative year. However, this variability has reduced and stabilized over time, especially since 2005 and more prominently since 2010. It is notable that the stabilization point for LN retrieval parallels the standardization of LN examination and acceptance of the importance of these assessments and the use of multidisciplinary approaches. Adequate LN evaluation is critical for staging and treating patients with colon cancer because the presence of LN metastases is one of the most important determinants of prognosis in these patients following curative resection and has a significant impact on whether adjuvant chemotherapy is recommended to a patient.^[2,3]^ There have been various studies on the number of nodes that need to be examined in order to accurately stage a colon cancer.^[4–7,16]^ Although this exact number remains unclear, a minimum of 12 LNs is recommended by many practice guidelines.^[2,17–19]^

Variable confounding factors may influence the level of lymph node retrieval. Patient, tumor, and treatment factors have been found to be associated with the number of retrieved lymph nodes in colon cancer. Surgical extent has been known influence on lymph node retrieved number.^[8,13]^ In our present study, surgical principles and the recommended extent according to the tumor location were maintained during the study period. In addition, the experience of the operators was both sufficient and standardized. Hence, we consider that the attending surgeon may be a possible confounding factor in LN retrieval but not the standardized surgical technique. The pathologic lymph node evaluation technique could influence the lymph node retrieval number but the same lymph node retrieval technique was used during the study period. We would expect therefore that the influence of the pathologic examination technique on lymph node retrieval would be mitigated.

In our present analyses, we found that the proportion of patients who had at least 12 LNs examined increased by a statistically significant extent between 1997 and 2013. This improvement is presumably due to multiple factors that have increased the awareness of the importance of LN examination for patients with colon cancer. We also found an increased multidisciplinary emphasis in the latter part of our study period on the importance of adequate LN evaluation during the curative resection of colorectal cancer.

Attention to the examined number of LNs in colon cancer has increased since 2000. This stemmed from findings that higher lymph node retrieval led to better oncological outcomes in patients with colon cancer. The initial studies focused on node-negative patients.^[20]^ That is, patients who had nodal disease that was undetected due to inadequate surgical resection or the failure of the pathologist to identify nodal metastasis. As a result of this inference, quality benchmarks regarding the number of LNs in a colectomy specimen have been proposed for surgeons, surgical techniques, and pathologic reviews. Nodal evaluation is likely to further improve with the recent development of the 12 LN standard proposed by multiple national oncology organizations. In one previous study using the National Cancer Data Base (NCDB), the proportion of hospitals that examined at least 12 LNs showed a considerable increase from 1996 to 2005.^[21]^

LN guidelines have facilitated better cooperation between surgeons and pathologists and raised awareness of other quality improvement issues. In our present study, an increase in the number of retrieved LNs was evident after the establishment of multidisciplinary treatment approaches at our hospital for colorectal cancer. When multidisciplinary approaches were applied, feedback was given between members of the team. The second plateau of recovered LNs in our study population could be a reflection of positive outcomes from this feedback. At our institution, the multidisciplinary approach has been used since 2005 and has become accepted practice over time. Although discussions on pathological examination results and the treatment of patients with colon cancer had taken place before the introduction of the multidisciplinary approach, it had not been systematized and had been limited to reviewing specific cases.

The beneficial role of multidisciplinary care in evaluating LNs has been described by studies that reported differences in LN evaluation between institutions.^[9,21]^ The review of the NCDB and Surveillance, Epidemiology, and End Results (SEER) data reported that the ability to achieve 12 LNs varied by institution. NCCN data revealed that the proportion of patients with >12 evaluated LNs was as high as 92%, in contrast to other hospitals included in SEER that achieved 12 LNs in only about 50% of patients. These differences also existed between high- and low-volume centers.^[14]^ The outcome measurement projects are well-known to many surgeons who practice at NCCN and high-volume centers. Because both surgeons and pathologists practice at these centers and are well-aware of the 12 LN benchmark, it is conceivable that this has contributed to a superior performance. A heightened sensitivity to the 12 LN benchmark at community hospitals with a less-dedicated cancer focus may also play a role in achieving the 12 LN metric in a greater number of patients.

Despite the best efforts of the surgeon and pathologist, other factors can still influence LN recovery. The tumor location may also be important. It is generally agreed that tumors on the right side are associated with higher numbers of examined LNs.^[10,15,22–24]^ Patients with left-sided colon cancer are known to be about 50% less likely to receive adequate LN evaluation.^[10,22]^ Indeed, the results of our present study reveal that LN evaluations differ by tumor location. Patients with transverse colon cancer and right colon cancer in our cohort did not differ in terms of the examined number of LNs. In comparing right-side and transverse colon cancer, however, specimens from the left side (descending and splenic flexure) and sigmoid colon had significantly fewer examined LNs. Age was also found to be related to the number of examined LNs. The proportion of our patients with <12 LNs also increased with age. Similar to previous studies that reported a low number of evaluated LNs in men,^[10,25]^ 89.3% of the female patients in our present study had ≥12 examined LNs. The tumor stage also demonstrated a significant association with the number of examined LNs. The proportion of patients with <12 examined LNs was higher in early-stage disease. However, surgeons may perform less-extensive operations on older patients and those with suspicious early-stage disease on preoperative diagnostics, and this factor may have confounded the association between age, stage, and number of evaluated LNs. However, the influence of these factors on LN retrieval was recently mitigated. These results and our current data together suggest that clinicopathological influences on LN retrieval could be overcome by a systematic approach and emphasis on the importance of LN retrieval.

Our present study had some limitations of note. Our analyses were retrospective study and thus susceptible to a selection bias. In addition, although the pathological lymph node method in our patient population was standardized and was unchanged during the entire study period, it could have variations with different examiners. Further, we used a subjective categorization of the study period according to the introduction of a multidisciplinary approach at our hospital in 2005, which influenced the surgical, oncological, and pathologic examinations performed. However, a certain proportion of our study patients, for example, patients with early colorectal cancer who were not candidates for adjuvant treatment did not have a chance to attend the multidisciplinary treatment clinic. Although, the multidisciplinary approach had an overall influence on the clinical treatment of the patients with colon cancer at our hospital, some patients did not benefit from discussions with the multidisciplinary team.

The importance of obtaining more LNs during colon cancer surgery is still controversial, and it is not universally accepted that examining more LNs will lead to better outcomes or improved staging accuracy.^[4,6]^ In addition, 12 LNs as a quality indicator of patient care remains the subject of debate. However, the process of LN examination during the curative resection of colon cancer has improved and the examination of at least 12 LNs has been achieved in nearly every case at our institution after adopting a multidisciplinary approach into standard practice. LN retrieval in colon cancer can be improved by better awareness and a more multidisciplinary emphasis on the importance of the number of examined LNs.
